# Efficacy and safety of pramlintide injection adjunct to insulin therapy in patients with type 1 diabetes mellitus: a systematic review and meta-analysis

**DOI:** 10.18632/oncotarget.16008

**Published:** 2017-03-08

**Authors:** Yong-Chao Qiao, Wei Ling, Yan-Hong Pan, Yin-Ling Chen, Dan Zhou, Yan-Mei Huang, Xiao-Xi Zhang, Hai-Lu Zhao

**Affiliations:** ^1^ Center of Diabetic Systems Medicine, Guangxi Key Laboratory of Excellence, Guilin Medical University, Guilin, China; ^2^ Department of Immunology, Xiangya School of Medicine, Central South University, Changsha, China; ^3^ Department of Immunology, Faculty of Basic Medicine, Guilin Medical University, Guilin, China

**Keywords:** tType 1 diabetes mellitus, pramlintide, postprandial glucose, adverse events, meta-analysis

## Abstract

**Aims:**

We aim to assess the efficacy and safety of pramlintide plus insulin therapy in patients with type 1 diabetes.

**Methods:**

We included clinical studies comparing pramlintide plus insulin to placebo plus insulin. Efficacy was reflected by glycemic control and reduction in body weight and insulin use. Safety concerns were hypoglycemia and other adverse events. Subgroup analysis was performed for different doses (30, 60, 90 µg/meal) and durations (≤4, 26, 29, >29 weeks) of the treatment.

**Results:**

A total of 10 randomized placebo-controlled studies were included for this meta-analysis (pramlintide, n=1978; placebo, n=1319). Compared with controls, patients given pramlintide had significantly lower HbA1c (*p* < 0.001), total daily insulin dose (*p* = 0.024), mean mealtime insulin dose (*p* < 0.001), body weight (*p* < 0.001) and postprandial glucose level (*p* = 0.002). The addition of pramlintide increased the incidence of nausea (*p* < 0.001), vomiting (*p* < 0.001), anorexia (*p* < 0.001) and hypoglycemia (*p* < 0.05) at the initiation of the treatment. The efficacy and adverse reactions of pramlintide were largely significant for the different doses and durations of the treatment.

**Conclusions:**

The addition of pramlintide to insulin therapy in patients with type 1 diabetes improves glycemic control and reduces insulin requirement and body weight while bringing transient hypoglycemia and digestive disorders.

## INTRODUCTION

Type 1 diabetes is characterized by the autoimmune destruction of the pancreatic islet β-cells with significant deficiency of the two glucose-modulating hormones, insulin and amylin [1]. Human amylin is a 37-amino acid peptide hormone which is synthesized by the pancreatic β cells and co-secreted with insulin in response to nutrient stimuli [2]. However, patients with insulin-dependent diabetes mellitus (type 1 diabetes) are amylin deficient [3, 4]. Human amylin is not likely to be used directly as a drug compound due to its physiochemical properties of poor solubility in aqueous milieu and self-aggregation into amyloid material [5, 6]. Instead, pramlintide, as an amylin agonist with improved water solubility to reduce the amyloid propensity [7], was approved by the US Food and Drug Administration in April 2005 for bolus premeal administration adjunct to insulin therapy in patients with diabetes mellitus [8]. Pramlintide differs from *de novo* human amylin by three amino acids but retains these pharmacodynamic properties as shown by improved glycemic control and weight loss [9]. Clinical studies have shown that mealtime subcutaneous pramlintide along with insulin, could regulate postprandial glucose appearance [10, 11], slow gastric emptying [12], suppress postprandial glucagon secretion [13], spare mealtime insulin use with benefit of overall weight loss [3, 14], reduce frequency of hemoglobin A1c (HbA1c) and food intake [15, 16] in both type 1 and type 2 diabetes [17-20]. Otherwise, hypoglycemia, nausea, vomiting and anorexia, the most frequently reported adverse events occurred early in the course of treatment and dissipated over time with continuation of therapy in patients receiving pramlintide, remained a key barrier for patients with diabetes and discrepancy existed between the studies which may be influenced by different doses and the durations of the treatment [20-22].

Because patients with type 1 diabetes require lifelong therapy and experience further deterioration of residual β-cells function, weight gain, and an increased risk of hypoglycemia over time after diagnosis, it is important to evaluate the effectiveness of treatment in patients across a spectrum of diabetes disease duration and pramlintide dose [18]. Taken together, the purpose of this meta-analysis is to assess the efficacy (e.g., reduction in HbA1c, postprandial glucose, and weight changes) and safety (risk of nausea, vomiting, anorexia and hypoglycemia) of combined use of pramlintide along with insulin in comparison with placebo treatment (placebo and insulin) in patients with type 1 diabetes in clinical randomized controlled trials.

## RESULTS

### The process and results of selection

**Figure 1:**
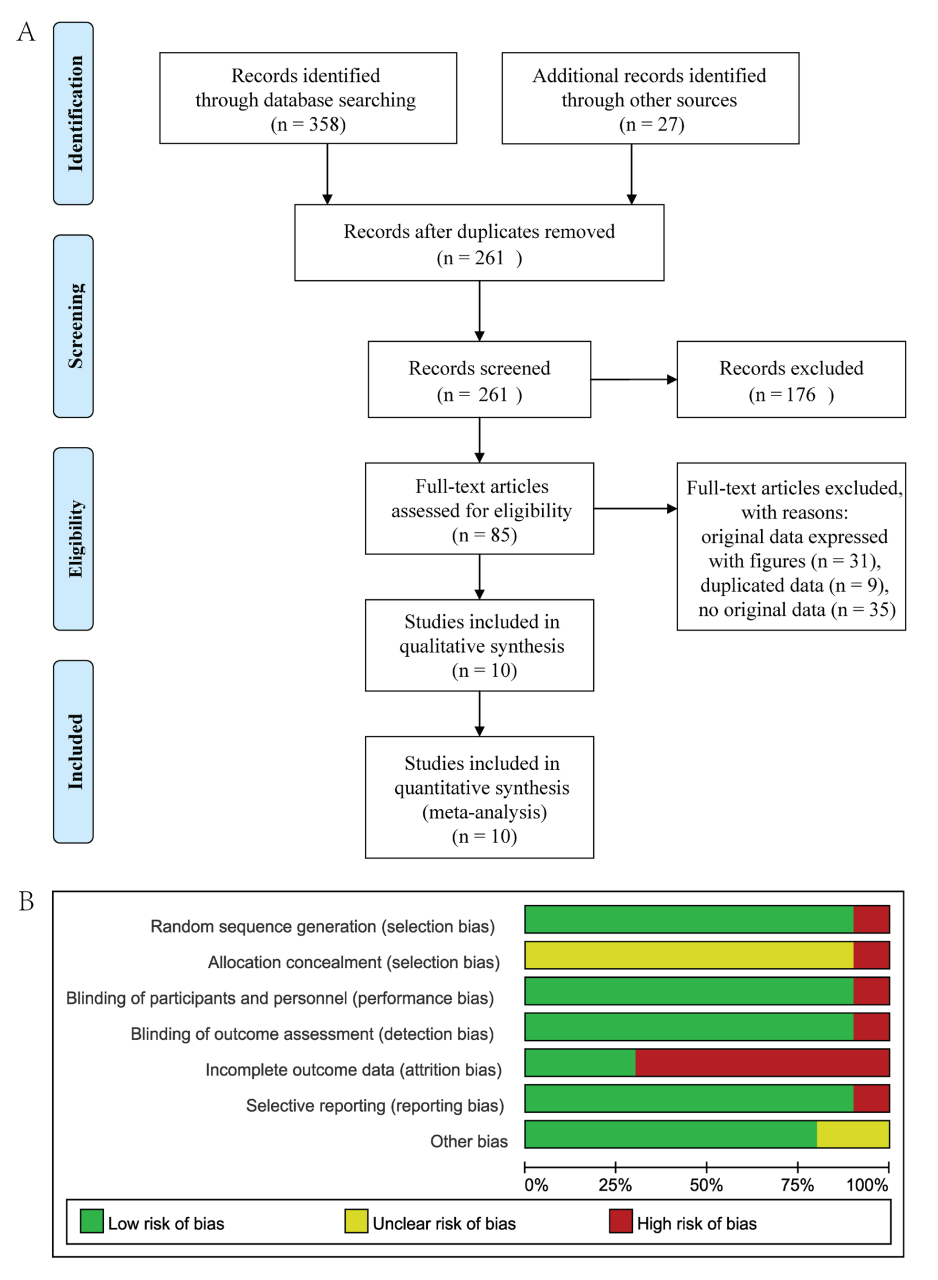
The flow chart of the studies search and inclusion process (**A**). Risk of bias in the included studies (**B**).

A total of 385 studies were recruited from the four databases by the search terms. Of these, 124 studies were excluded for reduplicative and 176 articles were eliminated after reviewing the title and abstract. Otherwise, 85 full-text studies were accessed and 75 studies were dropped according to the exclusion and inclusion criteria (Figure 1A). Of the 10 studies [3, 18, 20, 23-29] included in this meta-analysis (Pramlintide, n=1978; placebo, n=1319), six studies [3, 23-25, 28, 29] related both the efficacy and safety of the adjunctive therapy with pramlintide in type 1 diabetes mellitus and three [18, 20, 26] only focused on the safety and one [27] only tested the efficacy (Tables 1-2).

**Table 1:** The characteristics of studies about HbA1c, insulin dose and weight of patients between baseline and endpoint included in this meta-analysis

Author, year, country Reference	Study design	Pra dose (μg/meal)	HbA1c (Mean ± SD (%) or percent)		Insulin dose (Mean ± SE (%) or percent)		Weight (Mean ± SD (kg) or percent)	
			Baseline	Changed (%)	Baseline	Changed (%)	Baseline	Changed (%)
Edelman, S. 2006, USA [23]	Double-blind Placebo-controlled Randomized	30 or 60	Pra, 8.1±0.8, Pla, 8.1±0.8	At week 29§: Pra, -0.5 Pla, -0.5	Pra, 66.5±32.6, Pla, 63.7±32.4	At week 29: Pra, -28, Pla,-4	Pra, 81±17, Pla, 81±17,	At week 29§: Pra, -1.3±0.30, Pla, 1.2±0.24
Ratner, R. E. 2004, USA [3]	Double-blind Placebo-controlled Parallel-group Multicenter	Study1, 60TID, Study2, 60QID Study3, 90TID	Study1, 8.9±1.1, Study2, 8.9±1.0, Study3, 8.9±0.9, Pla, 9.0±1.1	At week 26:Pra, Study1*, -0.83, Study2*, -0.64, At week 52:Pra, Study1*, -0.59, Study2*, -0.57	NR	NR	Study1, 77.3±14.6, Study2, 78.3±14.5, Study3, 75.8±14.7, Pla,76.9±15.8	At week 26: Study 1‡, Pra, -1.3, Study 2‡, Pra, -0.8 Pla,0.7,
Marrero, D. G. 2007, USA [24]	Double-blind Placebo-controlled	30 or 60	Pra, 8.1±0.7, Pla, 8.1±0.8	At week 29NS: Pra, -0.39±0.07 Pla, -0.45±0.07	Pra, 55.7±28.8, Pla, 55.1±27.3	At week 29‡: Pra, -1.24±8.57 Pla, -4.44±3.39	Pra, 81.8±17.4, Pla, 80.6±17.0	At week 29§: Pra, -1.50±0.33, Pla, 1.28±0.25
Herrmann, K. 2016, USA [18]	Double-blind Placebo-controlled Post hoc analysis	30 or 60 QID	From 8.6±1.0 to 9.2±1.4	At week 26‡: (Pra, Pla) Study 1, -0.38,-0.09 Study 2, -0.51,-0.11 Study 3, -0.42, -0.14	From 57.3±44.7 to 45.5±26.3	At week 26*: (Pra, Pla) Study 1, 0.17,1.66, Study 2, -1.77, 0.35 Study 3, -2.12, 1.73	From 72.9±12.9 to 77.0±14.3	At week 26: (Pra, Pla) Study 1,-0.76,0.78 Study 2,-1.10, 0.56 Study 3,-1.24, 0.59
Herrmann, K. 2013, USA [20]	Double-blind Placebo-controlled Randomized Multicenter Post hoc analysis	30 or 60	Study 1, 8.1±0.7 Study 2, 8±1.1 Pla, 8±0.8	At week 29 NS: Pra, Study 1, -0.4, Study 2, -0.3, Pla, -0.3±0.1	Mealtime dose: From 18.5±10.7 to 25.7±14.3	At week 29: Pra, Study 1‡, -23.8±5.2, Study 2§, -27.5±2.9, Pla, -3.2±4.1	Study 1, 79±16 Study 2, 80±16 Pla, 80±17	At week 29: Study 1§, -2.2±0.5, Study 2§, -3.2±0.4, Pla, 1.4±0.3
Nyholm, B. 1999, USA [25]	Double-blind Placebo-controlled Randomized Crossover	30QID	All: 8.6±0.3	At week 4 NS: Pra, -0.7±0.3, Pla, -0.4±0.3	NR	NR	All: 74.9±2	At week 4 NS: Pra, -2.3±0.3, Pla, -1.3±0.4
Whitehouse, F. 2002, USA [26]	Double-blind Placebo-controlled Randomized Multicenter	30 or 60	Pra, 8.7±1.3, Pla, 8.9±1.5	At week 13§: Pra, -0.67, Pla, -0.16, At week 26‡: Pra, -0.58, Pla, -0.18, At week 52†: Pra, -0.39, Pla, -0.12	NR	At week 26*: Pra, 2.6, Pla, 9.5 At week 52*: Pra, 2.3, Pla, 10.3	Pra, 75.0±13.8, Pla, 75.6±13.3,	NR

Pra, pramlintide treated group; Pla, placebo treated group; TID, three times daily; QID, four times daily; NR, not report.

*, *p* < 0.05; †, *p* < 0.01; ‡, *p* < 0.001; §, *p* < 0.0001; NS, not significant

**Table 2:** The characteristics of studies about mean postprandial glucose and adverse events included in this meta-analysis

Author, year, country Reference	Subjects	Postprandial or fasting glucose	Nausea (%)	Vomiting (%)	Anorexia (%)	Hypoglycemia (event rate/patient-year or percent)
Edelman, S. 2006, USA [23]	Pra, 148 Pla, 147	NR	At week 29†: Pra, 62.8 Pla, 36.1	At week 29*: Pra, 13.5 Pla, 6.1	At week 29*: Pra, 8.8 Pla, 2.0	Pra vs. Pla At week 4NS: 0.75±0.25, 0.42±0.19 At week 29*: 0.57±0.09, 0.30±0.06
Ratner, R. E. 2004, USA [3]	Study 1, 164 Study 2, 161 Study 3, 172 Pla, 154	NR	At week 4: Pra, Study 1, 47.0 Study 2, 47 Study 3, 59 Pla, 12.0	At week 4: Pra, Study 1, 9.8 Study 2, 11 Study 3, 12 Pla, 6.5	At week 4: Pra, Study 1, 2.6 Study 2, 18.0 Study 3, 11 Pla, 16	Pra vs. Pla (Study 1, 2, 3, Pla) At week 4: 3.78±0.57, 3.41±0.55, 3.91±0.58, 0.87±0.27 At week 26: 1.13±0.15, 0.98±0.13, 0.96±0.14, 0.80±0.12 At week 52: 0.74±0.12, 0.79±0.12, 0.64±0.12, 0.45±0.09
Herrmann, K. 2016, USA [18]	Study 1, Pra, 223, Pla, 192, Study 2, Pra, 243, Pla, 176, Study 3, Pra, 248, Pla, 169	NR	At week 26: Study 1, Pra, 35, Pla, 13.5 Study 2, Pra, 46.9, Pla, 17.0 Study 3, Pra, 53.2, Pla, 15.4	At week 26: Study 1, Pra, 6.3, Pla, 5.2 Study 2, Pra, 10.3, Pla, 8.0 Study 3, Pra, 11.7, Pla, 4.1	At week 26: Study 1, Pra,5.8, Pla, 1.6 Study 2, Pra,7.4, Pla, 1.1 Study 3, Pra, 10.9, Pla, 0.6	At week 26: Study 1, Pra, 13.5, Pla, 9.4 Study 2, Pra, 21.8, Pla, 15.3 Study 3, Pra, 27.4, Pla, 21.9
Herrmann, K. 2013, USA [20]	Study 1, 67, Study 2, 100, Pla, 67	NR	At week 29: Pra, Study 1, 67.1, Study 2, 43.3, Pla, 37	NR	NR	At week 29: Study 1, Pra, 56, Study 2, Pra, 12, Pla, 34
Whitehouse, F. 2002, USA [26]	Pra, 243 Pla, 237	NR	At week 52: Pra, 46.5 Pla, 21.9	At week 52: Pra, 11.5 Pla, 8.0	At week 52: Pra, 17.7 Pla, 2.1	Pra vs. Pla At week 4: 2.12±0.35, 2.00±0.34 At week 26: 0.74±0.09, 1.37±0.13 At week 52: 0.43±0.07, 1.24±0.12
Thompson, R. G. 1997a, USA [27]	Study 1, 38, Study 2, 39 Study 3, 36, Study 4, 40 Pla, 38	Changed to week 4: (mmol/l) Pra, Study 1, -1.4±0.5 Study2, -0.03±0.5 Study 3, -0.1±0.4 Study 4, -0.9±0.5 Pla, 0.3±0.5	NR	NR	NR	At week 4: Pra, Study 1, 12.2, Study 2, 22.2 Study 3, 25, Study 4, 20.9 Pla, 26.2
Thompson, R. G. 1997b, USA [28]	Study 1, 40, Study 2, 40, Study 3, 40, Pla, 39	Changed to week 2: (mmol/l) Pra, -1.9±0.4, Pla, -0.03±0.5	At week 2: Pra, Study 1, 2.3, Study 2, 19.5 Study 3, 42.9, Pla, 2.4	NR	At week 2: Study 1, Pra, 0, Study 2, 2.4 Study 3, Pra, 9.5, Pla, 0	At week 2: Pra, Study 1, 79.1 Study 2, 85.4 Study 3, 81, Pla, 81
Weinzimer, S. A. 2012, USA [29]	Pra, 8 Pla, 8	At week 29: (mg/dl) Pra, 88±42, Pla, 113±32	NR	NR	NR	NR
Marrero, D. G. 2007, USA [24]	Pra, 130 Pla, 136	At week 29: (mg/dl) Pra, 151.3±2.2, Pla, 172.7±2.1	NR	NR	NR	NR

Pra, pramlintide treated group; Pla, placebo treated group; TID, three times daily; QID, four times daily; NR, not report.

*, *p* < 0.05; †, *p* < 0.01; NS, not significant

All of the data sets included in the meta-analysis were from randomized placebo-controlled clinical trials. Figure 1B shows the high methodological quality of the trials and low risks of bias in the included studies.

### Pramlintide adjunct to insulin reduced HbA1c

**Figure 2:**
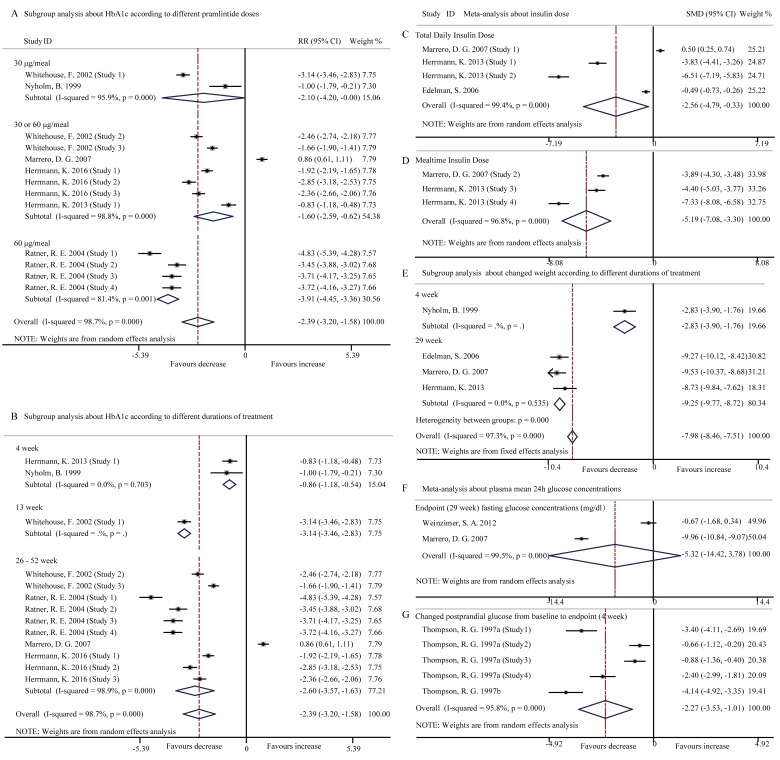
Forest plots for the level of HbA1c, insulin dose, changed body weight and glucose concentration between pramlintide treated and placebo treated patients with T1DM **A.**, Subgroup analysis about the level of HbA1c according to different pramlintide doses (30 μg/meal, *p* = 0.05; 30 or 60 μg/meal, *p* = 0.001; 60μg/meal, *p* < 0.001; overall, *p* < 0.001); **B.**, Subgroup analysis about the level of HbA1c according to different durations of treatment (4 week, *p* < 0.001; 13 week, *p* < 0.001; 26-52 week, *p* < 0.001; overall, *p* < 0.001); **C.**, Total insulin dose (*p* = 0.024); **D.**, Mealtime insulin dose (*p* < 0.001); **E.**, Subgroup analysis about changed body weight according to different durations of treatment (4 week, *p* < 0.001; 29 week, *p* < 0.001; overall, *p* < 0.001); **F.**, Fast glucose concentrations at 29 week endpoint (*p* = 0.252); G, Changed postprandial glucose from baseline to endpoint (*p* = 0.002).

Pooled data with the random-effects model analysis from the included studies [3, 18, 20, 24-26] displayed that treatment with pramlintide led to a significant reduction in HbA1c (SMD, -2.39; 95% CI, -3.20 to -1.58; *p* < 0.001) with high heterogeneity between studies (*I*2 = 98.7%, *p* < 0.001) (Figure 2A, 2B). The maximal reduction in HbA1c (maximum 0.41%) by pramlintide was observed within 26 weeks of the treatment, from baseline in the 60-μg TID 0.41% (*p* = 0.012) compared with the 0.18% reduction in the placebo group [3]. Among the six included studies, five were consistent with the outcome of the meta-analysis and only one showed a similar reduced mean HbA1c in two groups (pramlintide: -0.39±0.07%; placebo: -0.45±0.07%) [24].

Subgroup analysis was performed to explore the impact of different pramlintide doses and durations of treatment for the high heterogeneity and that significant reduction in HbA1c level was found in 30 μg/meal, 30 or 60 μg/meal, and 60μg/meal pramlintide treatment groups (all *p* < 0.05) (Figure 2A). Reduction in HbA1c was also significant in all the three durations (4 weeks, 13 weeks and 26-29 weeks; all *p* < 0.001) of the pramlintide treatment (Figure 2B). Subgroup analysis disclosed that the doses and durations of the treatment had impacts on HbA1creduction. Single factor regression analysis indicated that pramlintide dose (*t* = 0.13, *p* = 0.089, 95% CI, -2.608 to 2.940) and duration of treatment (*t* = -1.30, *p* = 0.221, 95% CI, -3.823 to 2.811) were not the source of the high heterogeneity.

### Pramlintide adjunct to insulin reduced insulin dose

Pooled data from the three studies [20, 23, 24] disclosed that pramlintide-treated patients had also significantly reduced both total daily insulin dose (SMD, -2.56; 95% CI, -4.79 to -0.33; *p* = 0.024) (Figure 2C) and mean mealtime insulin dose (SMD, -5.19; 95% CI, -7.08 to -3.30; *p* < 0.001) (Figure 2D) in the random-effects model analysis with high heterogeneity (*I*2 = 99.4%, *p* < 0.001; *I*2 = 96.8%, *p* < 0.001 respectively). “Pramlintide dose escalation with concomitant insulin dose reduction during initiation lowered rates of adverse events in pramlintide-treated patients to levels similar to placebo-treated patients using insulin” [23].

### Pramlintide adjunct to insulin reduced body weight

Four studies [20, 23-25] assessed changes in body weight and meta-analysis of the studies showed that pramlintide significantly decreased body weight from baseline to endpoint (SMD, -7.98; 95% CI, -8.46 to -7.51; *p* < 0.001) (Figure 2E). After excluding a 4-week trial, the finding of pramlintide-induced weight loss (*p* < 0.001) was not changed but the heterogeneity disappeared (*I*2 = 0, *p* = 0.535). Subgroup analysis revealed a trend of body weight reduction in parallel to prolonged durations of the pramlintide treatment.

### Pramlintide adjunct to insulin reduced postprandial glucose level

Treatment with pramlintide for 29 weeks lowered fasting glucose level without statistical significance (SMD, -5.32; 95% CI, -14.42 to 3.78; *p* = 0.252) in the random-effects model analysis (*I*2 = 99.5%, *p* < 0.001) (Figure 2F), based on the pooled data from the two studies [24, 29]. In contrast, the other two studies [27, 28] consistently reported a significantly reduced plasma mean 24h postprandial glucose level (SMD, -2.27; 95% CI, -3.53 to -1.01; *p* < 0.001) (Figure 2G), based on the random-effects model analysis (*I*2 = 95.8%, *p* < 0.001). Pramlintide reduced postprandial glucose rather than fasting glucose [24], a therapeutic benefit consistent with the pharmacodynamic profile of pramlintide.

### Pramlintide adjunct to insulin induced digestive disorders and hypoglycemia

**Figure 3:**
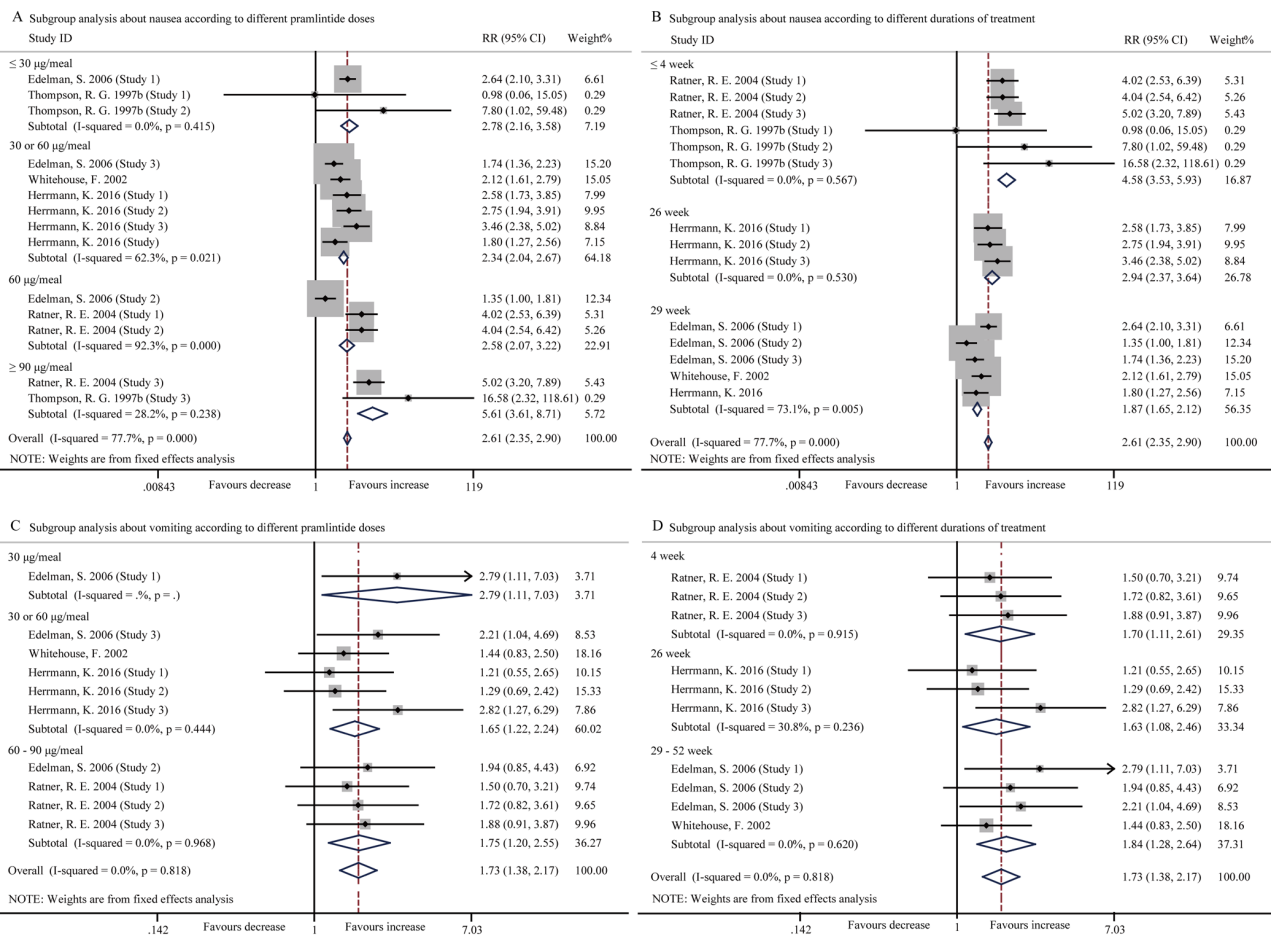
Forest plots for the incidence of nausea and vomiting between pramlintide treated and placebo treated patients with T1DM **A.**, Subgroup analysis about the incidence of nausea according to different pramlintide doses (30 μg/meal, *p* < 0.001; 30 or 60 μg/meal, *p* < 0.001; 60 μg/meal, *p* < 0.001; ≥ 90 μg/meal, *p* < 0.001; overall, *p* < 0.001); **B.**, Subgroup analysis about the incidence of nausea according to different durations of treatment (4 week, *p* < 0.001; 13 week, *p* < 0.001; 29 week, *p* < 0.001; overall, *p* < 0.001); **C.**, Subgroup analysis about the incidence of vomiting according to different pramlintide doses (30 μg/meal, *p* = 0.030; 30 or 60 μg/meal, *p* = 0.001; 60-90 μg/meal, *p* = 0.004; overall, *p* < 0.001); **D.**, Subgroup analysis about the incidence of vomiting according to different durations of treatment (4 week, *p* = 0.014; 26 week, *p* = 0.021; 29-52 week, *p* = 0.001; overall, *p* < 0.001).

**Figure 4:**
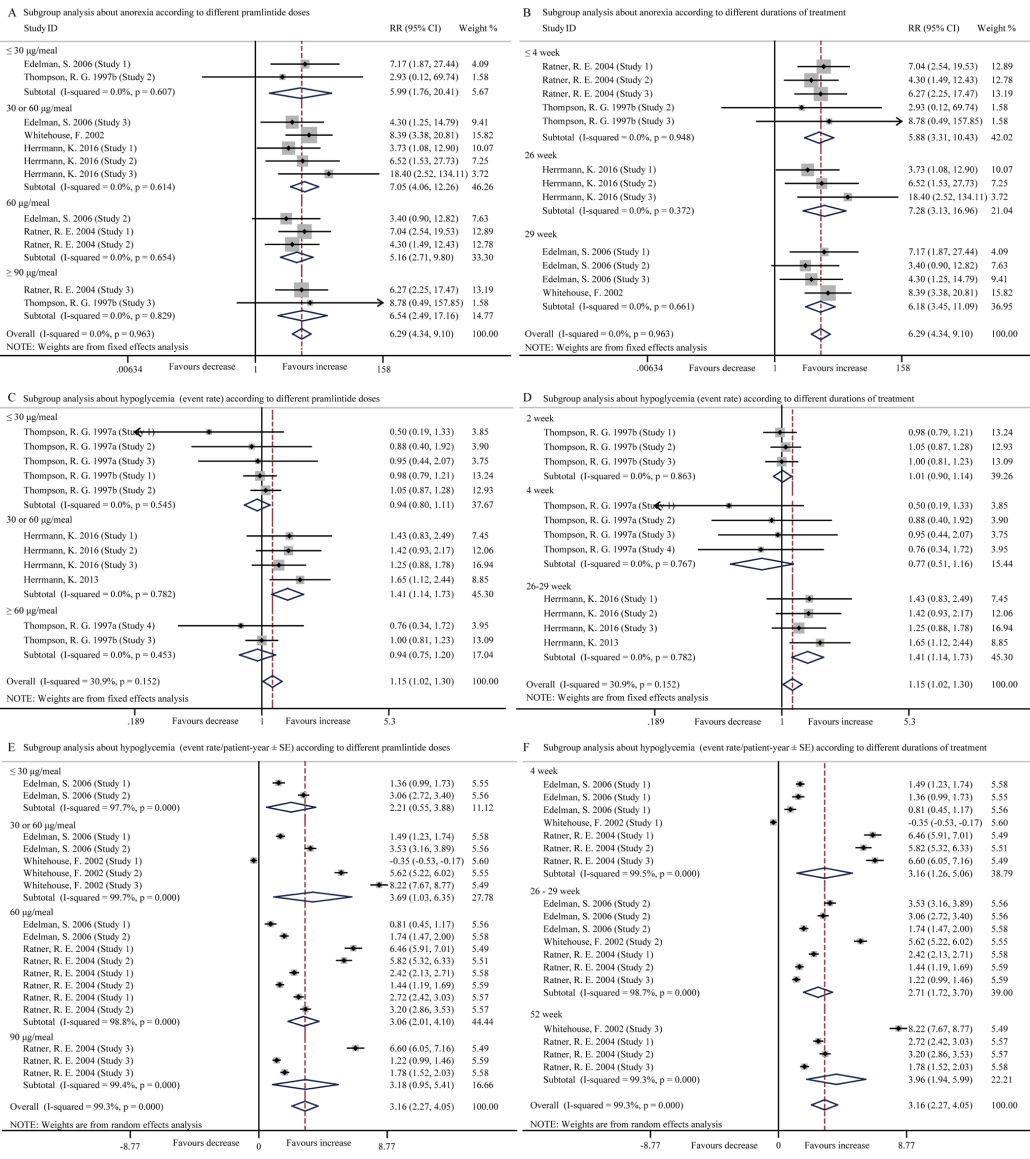
Forest plots for the incidence of anorexia and hypoglycemia (event rate) between pramlintide treated and placebo treated patients with T1DM **A.**, Subgroup analysis about the incidence of anorexia according to different pramlintide doses (≤ 30 μg/meal, *p* = 0.004; 30 or 60 μg/meal, *p* < 0.001; 60 μg/meal, *p* < 0.001; ≥ 90 μg/meal, *p* < 0.001; overall, *p* < 0.001); **B.**, Subgroup analysis about the incidence of anorexia according to different durations of treatment (≤ 4 week, *p* < 0.001; 26 week, *p* < 0.001; 29 week, *p* < 0.001; overall, *p* < 0.001); **C.**, Subgroup analysis about the incidence of hypoglycemia (event rate) according to different pramlintide doses (≤ 30 μg/meal, *p* = 0.483; 30 or 60 μg/meal, *p* = 0.001; ≥ 60 μg/meal, *p* = 0.635; overall, *p* = 0.025); **D.**, Subgroup analysis about the incidence of hypoglycemia (event rate) according to different durations of treatment (2 week, *p* = 0.867; 4 week, *p* = 0.215; 26-29 week, *p* = 0.001; overall, *p* = 0.025); **E.**, Subgroup analysis about incidence of hypoglycemia (event rate/patient-year ± SE) according to different pramlintide doses (≤ 30 μg/meal, *p* = 0.009; 30 or 60 μg/meal, *p* = 0.006; 60 μg/meal, *p* < 0.001; 90 μg/meal, *p* = 0.005; overall, *p* < 0.001); **F.**, Subgroup analysis according to different durations of treatment (4 week, *p* = 0.001; 26-29 week, *p* < 0.001; 52 week, *p* < 0.001; overall, *p* < 0.001).

Hypoglycemia and digestive disorders such as nausea, vomiting, and anorexia were the main safety concerns of pramlintide injection. In our study, compared with the control, pramlintide induced significantly higher incidence rates of nausea (RR, 2.61; 95% CI, 2.35 to 2.90, *p* < 0.001) (Figure 3A, 3B), vomiting (RR, 1.73; 95% CI, 1.38 to 2.17, *p* < 0.001) (Figure 3C, 3D), anorexia (RR, 6.29; 95% CI, 4.34 to 9.10, *p* < 0.001) (Figure 4A, 4B) and hypoglycemia (Event rate, RR, 1.15; 95% CI, 1.02 to 1.30, *p* = 0.025 (Figure 4C, 4D); Event rate/patient-year, SMD, 3.16; 95% CI, 2.27 to 4.05, *p* < 0.001 (Figure 4E, 4F)). High heterogeneity existed in the pooled data about nausea (*I*2 = 77.7%, *p* < 0.001), but not vomiting (*I*2 = 0.0%, *p* = 0.818), anorexia (*I*2 = 0.0%, *p* = 0.963) and hypoglycemia (Event rate, *I*2 = 30.9%, *p* = 0.152).

Subgroup analysis indicated that the adverse events of nausea, vomiting, anorexia and hypoglycemia (Event rate/patient-year) occurred more frequently in the entire pramlintide-treated subgroups (all *p* < 0.05) according to different pramlintide doses and durations of treatment (Figure 3, 4). However, changes of incident hypoglycemia by event rates were not significant with the doses of ≤ 30 μg/meal (Event rate, RR, 0.94; 95% CI, 0.80 to 1.11, *p* = 0.483) and ≥ 60 μg/meal (Event rate, RR, 0.94; 95% CI, 0.75 to 1.20, *p* = 0.635) and with the durations of 2-week (RR, 1.01; 95% CI, 0.90 to 1.14, *p* = 0.867) and 4-week (RR, 0.77; 95% CI, 0.51 to 1.16, *p* = 0.215) (Figure 4C, 4D). Otherwise, high heterogeneity was significantly reduced in all pramlintide dose subgroups and all different durations of treatment subgroups (Figure 3A, 3B), which may explain the origin of the high heterogeneity in the incidence of nausea.

Single factor regression analysis indicated that significant difference in the incidence of hypoglycemia (Event rate) existed among the subgroups analysis related to the pramlintide doses (*t* = 4.54, *p* = 0.001, 95% CI, 0.511 to 1.527) and durations of treatment (*t* = 7.72, *p* < 0.001, 95% CI, 0.570 to 1.042).

Pramlintide therapy was generally well tolerated and there was no evidence of cardiovascular, pulmonary, hepatic, or renal toxicity associated with its use [20]. The most common adverse event, other than hypoglycemia, was nausea which was generally mild to moderate in intensity and no vomiting and anorexia events appeared in RCT trials [20]. Otherwise, no episodes of hypoglycemia existed and none of the subjects reported nausea, abdominal pain, bloating, distension, diarrhea, headache, or any other symptoms in response to pramlintide administration [29]. Lack of original data prevented this meta-analysis.

### Sensitivity analysis

Sensitivity analysis was implemented to evaluate the results and we found all of the results remained relatively stable by excluding individual studies or changing the Cochran’s Q statistic methods.

### Publication bias

Egger’s test proved that no significant bias existed in the pooled data of the level of changed HbA1c (*t* = 1.85, *p* = 0.091, 95%CI, -2.729 to 31.892), insulin dose (Total daily insulin: *t* = -4.06, *p* = 0.056, 95%CI, -52.609 to 1.522; Mealtime insulin: *t* = -1.62, *p* = 0.352, 95%CI, -134.294 to 103.885), body weight (*t* = 1.14, *p* = 0.372, 95%CI, -75.832 to 130.588) and the adverse events of anorexia (*t* = 0.55, *p* = 0.595, 95%CI, -7.547 to 12.479) and hypoglycemia (Event rate: *t* = 0.43, *p* = 0.679, 95%CI, -1.533 to 2.247) except the changed postprandial glucose (*t* = -14.59, *p* = 0.001, 95%CI, -25.942 to -16.649), nausea (*t* = 2.76, *p* = 0.017, 95%CI, 1.877 to 15.851), vomiting (*t* = 2.40, *p* = 0.043, 95%CI, 0.241 to 12.290) and hypoglycemia (Event rate/patient-year: *t* = 8.61, *p* < 0.001, 95%CI, 27.247 to 45.051).

## DISCUSSION

The results of this meta-analysis indicate that pramlintide supplement as an adjunct to insulin therapy might improve glycemic control and reduce body weight and insulin dose while bring transient hypoglycemia and digestive disorders in patients with type 1 diabetes. The outcomes of this study are consistent with the pharmacodynamic profile of pramlintide, including attenuated diurnal and postprandial glycemic excursions, enhanced satiety, reduced food intake [4, 10, 11], and triggered the risk of adverse events. On the other hand, in the placebo-controls, it is not attainable to allow patients to achieve metabolic improvement by insulin-treated alone. Pramlintide, with a mechanism of action that complementary to the insulin, offers such an alternative and is the only anti-diabetes therapy other than insulin which is approved for use in patients with T1DM [20].

Prior clinical trials showed that pramlintide-treated patients had significantly reduced HbA1c, postprandial glucose concentrations, mealtime insulin dose and body weight [18, 20, 23]. A meta-analysis also proved that pramlintide was somewhat more effective than placebo as an adjunct therapy for improving HbA1c levels and weight in adults with T1DM [30]. Chase, H. P., *et al.* [31] proved that pramlintide obviously reduced postprandial glucagon and glucose excursions and slowed gastric emptying in adolescents with type 1 diabetes. Fineman, M. S., *et al.* [32] concluded that mealtime amylin replacement with pramlintide prevented the abnormal meal-related rise in insulin-treated T1DM patients with glucagonemia. Hinshaw, L., *et al.* [17] demonstrated that inhibition of glucagon secretion with delayed gastric emptying reduced 2-hour prandial glucose excursions in T1DM by postponing meal rate of glucose appearance. The improvement in postprandial blood glucose control by pramlintide was coupled with a significant reduction in mealtime insulin dose [20, 24] which might be relevant due to the cardiovascular risk of high insulin doses [33], and it is consistent with the complementary actions of amylin. Postprandial glucose control is clinically significant for two reasons. First, it is an important component of overall glycemic exposure (HbA1c) [23]. Second, independent of its effect on the HbA1c, postprandial hyperglycemia has been implicated in the development of micro- and macro-vascular complications [23]. The potential advantage of pramlintide as an adjunct to insulin therapy is the ability of insulin pumps to deliver mealtime insulin boluses over an extended period of time. Because pramlintide slows gastric emptying, and therefore carbohydrate absorption, delaying at least a portion of the insulin bolus by using these features may result in improved post-meal blood glucose control [20]. Previous study has shown the data supporting an action of amylin agonist by decreasing gastrict emptying and thereby reduce the rate of peak postprandial carbohydrate absorption which may provide an advantage to using insulin and pramlintide in closed-loop systems compared with insulin alone [29].

However, Marrero, D. G., *et al.* [24] followed 29 weeks of pramlintide treatment in T1DM patients and concluded that postprandial glucose excursions were significantly reduced in pramlintide-treated patients while having equivalent overall glycemic control as placebo-treated patients, as measured by mean postprandial glucose concentrations. In addition, pramlintide treatment was associated with reductions in mean body weight and mealtime insulin use over 29 weeks but that mean HbA1c was reduced to a similar degree in both pramlintide- and placebo-treated patients. Difference in pramlintide dosages and durations of treatment may significantly influence the outcomes, and the treatment about mealtime insulin dose (multiple daily injection or continuous subcutaneous insulin infusion pump therapy) may explain the inconformity. Weight gain is not only a frequent cosmetic deterrent to intensification that is known to lead to non-adherence to insulin therapy [34], but can also correlate with various components of the metabolic syndrome [35], predicting increased cardiovascular risk in overweight patients with T1DM [33]. Therefore the therapies of improving glycemic control and maintaining or reducing body weight are increasingly important for T1DM patients [20]. Immunologic modulation of amylin and amylin analogs in diabetes warrant future investigation [36].

The increased risk of severe hypoglycaemia is another concern for improving glycaemic control with insulin therapy in T1DM patients. Fear of severe hypoglycaemia deters both patients and physicians from striving for better glycaemic control [37]. Based on this respect, pramlintide does not cause hypoglycaemia in healthy individuals as anti-hyperglycaemic agent, even at very high doses [3]. This indicates that in a clinical setting an increased initial risk of severe hypoglycaemia should be avoidable with careful blood glucose monitoring and judicious insulin dose-adjustment, particularly at mealtime [3]. Otherwise, the actions elicited by pramlintide, namely delayed gastric emptying, reduced food intake, and postprandial glucose reduction were an recipe for increased risk of hypoglycemia [18].

Pramlintide therapy is generally well tolerated. Nausea, anorexia and vomiting are the most common adverse events associated with pramlintide therapy which are mostly of mild to moderate intensity. Generally, the adverse events are dose-dependent, occurred early in the course of the treatment and dissipated over time with continuation of therapy, but in our study, the increased incidence of adverse events occurred in all the different doses and durations of the treatment. The small number of studies and the small sample of subjects included may result in the safety concerns. In any case, adverse events inevitably happened in T1DM patients with pramlintide therapy and the reason of the adverse events is unclear, although a combination of delayed gastric emptying, reduced caloric intake and inadequate adjustment to insulin therapy have been suggested [9]. Previous study reported that the largest improvements in HbA1c levels and weight occurred during the initial 6 months of treatment and then deteriorated with time [30]. Otherwise, there are no trials that evaluated long-term health outcomes and adverse events to determine whether benefits outweigh risks, and few studies are published on patient reported outcomes [30]. Although the risk of adverse events is higher with pramlintide than placebo in this study, the access of good-quality, long-term evidence about the effects of pramlintide is insufficient and larger RCTs with follow-up longer than 1 year are needed. So the appropriate and fixed dose of pramlintide may depend on the clinical conditions of the patients.

Limitations should be considered when reading the findings. Adequate methods about reporting randomization and allocation concealment were missing and unclear approaches of double blinding existed in some collected studies. Otherwise, publication bias inevitably existed in some meta-results, whereby studies with positive results are more likely to be published than negative studies.

In summary, the present meta-analysis indicates that pramlintide treatment as an adjunct to insulin therapy in T1DM patients has significantly improved glycemic control, reduced insulin dose and decreasing body weight, while showing nausea, vomiting, anorexia and hypoglycaemia in the initial titration phase.

## MATERIALS AND METHODS

### Search strategies

This study was performed according to the Preferred Reporting Items for Systematic Reviews and Meta-analysis (PRISMA) criteria [38]. Ethical approval was obtained from the local Research Ethics Committee. Relevant studies were searched from the database of Pubmed, Medline, Cochrane Library and EBSCOhost databases from inception until December 2016. The retrieval strategy of ‘‘pramlintide or amylin analogue” and “diabetes or type 1 diabetes mellitus or T1DM or diabetes mellitus or DM” was conducted and all the additional reports were retrieved manually through references cited in recruited articles for further evaluation.

### Inclusion criteria and exclusion criteria

Studies included should meet all the following criteria: (1) Study design was randomized controlled trials; (2) Subjects were patients with type 1 diabetes; (3) Pramlintide was used as an adjunct to insulin therapy while placebo-treated patients were defined as controls; (4) Patients were free from complications; and (5) The results of the study should include changes of HbA1c, insulin dose, body weight, postprandial glucose or adverse events such as nausea, vomiting, anorexia and hypoglycemia.

Exclusion criteria: (1) Animal studies, reviews, case reports, and personal experience summaries; (2) Only the latest paper was included into our final analysis related to duplicated studies and reports; (3) Original data displayed as figures or no original data reported; and (4) Inconsistent with the inclusion criteria as described above.

### Quality assessment

The risk of bias in the included studies was assessed by the criteria described in the Cochrane Handbook through the tool of Review manager software 5.1 [39]. Items included random sequence generation (Selection bias), allocation concealment (Selection bias), blinding of participants (Performance bias), personnel and assessors of outcomes (Detection bias), incomplete data on outcomes (Attrition bias), selective reporting (Reporting bias) and funding by industry (Other bias). A judgment of ‘Low risk’ of bias, ‘High risk’ of bias, or ‘Unclear risk’ of bias was assessed by two review authors independently, which is followed by a text box for a description of the above items that underlie the judgment. The contradiction was resolved through discussion.

### Data extraction

Details of the published studies were reviewed and extracted by two investigators independently as follows: (1) first author’s name; (2) date of publication; (3) country of the studied population; (4) study design; (5) sample size of controlled and experimental group; (6) Mean ± SD of HbA1c, insulin dose, weight or postprandial glucose; (7) participants number and incidence of adverse events such as nausea, vomiting, anorexia or hypoglycemia. If disagreements existed between the two investigators during data extraction, the third investigator was invited to assess such articles through discussion.

### Statistical analysis

Primary analyses assessed the continuous data about the changed HbA1c, insulin dose, body weight and postprandial glucose from baseline to endpoint between experimental and controlled groups. Binary data sets about adverse events such as nausea, vomiting, anorexia and hypoglycemia were compared between the two groups at the endpoint time. Otherwise, subgroup analysis was analyzed based on different pramlintide doses and durations of treatment. Chi-squared Q test and I2 statistics were used to estimate the heterogeneity [40, 41]. When *p* < 0.1 or I2 > 50%, we selected a random-effect model to account for possible heterogeneity between studies; otherwise a fixed-effect model was used in the absence of significant heterogeneity [42]. Single factor regression analysis was used to explore sources of heterogeneity. Sensitivity analysis was performed by excluding individual studies or changing the Cochran’s Q statistic methods to check the stability of the results. Publication bias was quantified by an Egger’s test (*p* < 0.05 was considered representative of statistically significant publication bias) [43]. The statistics were performed using the Stata 12.0 software and the risk of bias assessment was presented as graphs using Review Manager Version 5.1.
